# Electronic Medical Consultation: A New Zealand Perspective

**DOI:** 10.2196/jmir.3.1.e13

**Published:** 2001-03-17

**Authors:** Campbell Brebner, Raymond Jones, Wendy Marshall, Graham Parry, Alec Holt, Jayne Krisjanous

**Affiliations:** ^1^HealthInformatics GroupUniversity of OtagoWellington School of MedicineWellingtonNew Zealand

**Keywords:** Electronic Medical Consultation, New Zealand, Online Health

## Abstract

Electronic medical consultation is available worldwide through access to the World Wide Web (WWW). This article outlines a research study on the adoption of electronic medical consultation as a means of health delivery. It focuses on the delivery of healthcare specifically for New Zealanders, by New Zealanders. It is acknowledged that the WWW is a global marketplace and that it is therefore difficult to identify New Zealanders' use of such a global market; nevertheless, we attempt to provide a New Zealand perspective on electronic medical consultation.

## Introduction

Electronic medical consultation as a means of health delivery is available worldwide. Although only in its infancy in New Zealand, it is likely to gain momentum and acceptance and will have an impact on both the health deliverer and the consumer. The adoption of electronic consultation could radically change the environment of healthcare. The emergence of new business models and social impacts are just two of the areas where there could be significant change.

As technology is embraced by commercial, health, and other interests, law and governance are left struggling to keep up with the changes. Will the gap between the "haves" and "have-nots" widen or close? Has a beast been unleashed, or are we embarking into a brave new world where anyone can access the health information they need, regardless of socio-economic status, race, or geographic situation? We discuss these questions with an emphasis on the New Zealand scene.

## Discussion

### Background

Traditionally, patients and health providers have interacted face to face. The arrival of the telephone revolutionized communication, yet it did not significantly alter the way health providers and patients interact. The introduction of the Internet into the public arena throughout the 1990s has paved the way for significant advances in communication and information exchange in the health industry. The facility of e-mail, via the Internet, allows for the quick and efficient transmission of a written message to a targeted receiver. This article predicts profound alterations in the healthcare infrastructure, changes that will provide exciting opportunities at all levels of healthcare, from individual providers to large multinational corporation initiatives.

E-mail consultation offers patients numerous opportunities, including convenience, the ability to access second opinions, and the ability to choose from a wide range of specialists who might otherwise have been inaccessible. Jones suggested that although there are many concerns about the rise of "Web doctors," the number of them is likely to increase [1].

A study analyzing requests for consultations at a free pediatric e-mail consultation service for parents gave rise to the following conclusions: (1) Parents would rather use e-mail than face a "harassed" doctor for further explanations. (2) Parents were not overly concerned about posting personal details that may not be secure. The authors concluded that e-mail was a legitimate way for patients to receive disease-specific information in a timely manner [2]. In addition, many patients apparently find it difficult to discuss embarrassing or taboo subjects with their doctors. Howe reported that this anonymous, faceless form of consultation can be at once personalized and anonymous [3].

Dr Mulholland, a general practitioner based in Taranaki, New Zealand, operates a commercial e-health service called "doctorglobal," which is reported to be outstandingly successful [4]. This type of enterprise is gaining the attention of professional medical associations, which believe that some standards and protocols should be set [5]. Conversely, commenting on the launch of "doctorglobal," Dr Wiles, chairman of the New Zealand College of General Practitioners, described e-mail consultation as "dangerous nonsense" [4]. However, there are publicly funded initiatives in New Zealand that are taking advantage of the possibilities offered by e-health. These initiatives include the Waikato Tele-dermatology, the Waitemata Tele-psychiatry, the South Island Tele-medicine Project, the Christchurch Tele-medicine Service, and the New Zealand Tele-paediatric Service [6].

In researching this article we found that positions on the future impact and appropriateness of telemedicine seem to be polarized. At one pole are the "tele-evangelists," who believe that telemedicine will lead to a more patient-focused model. At the other pole are the "tele-Luddites," who think that telemedicine introduces technology that complicates an already complex healthcare environment and that will always come second to face-to-face interactions [7].

### Social Implications

The arrival of e-commerce has caught the health sector unprepared and without existing conventions. Innovators have adopted electronic medical consultation at a pace that has surpassed the formalization of any frameworks or guidelines. This situation has engendered a developmental environment that is relatively unfettered by any of the standards usually applied to a new form of treatment or service.

It is likely that protocols and guidelines will evolve as emerging trends and patterns become more obvious or pressing. However, because of a significant gap in the literature related to social impacts (particularly empirical work), predictions put forward continue to be speculation. There are several relevant potential social effects of e-health, including issues related to equity, consumerism, and altered relationships.

#### Equity

Most national health systems should develop equity policies for electronic medical consultation. Although Internet connections are very accessible in New Zealand, computing resources should be made available to the consumer to ensure equity. The profile of the Internet-enabled consumer is significantly skewed to higher socio-economic and better educated segments [8,9]. Therefore, patient-initiated e-mail consultation may have entry barriers. As well as access to resources, the user must have adequate language abilities, literacy, and technical knowledge. These requisites give several groups, such as ethnic minorities, older persons, the poor, and people with literacy problems, a potential equity disadvantage with regard to electronic healthcare options. Some of these may be within. Eysenbach highlighted this point in his discussion of a potential widening gap between privileged "Internet-able" populations and underprivileged populations that will not be able to participate in Internet-distributed healthcare [10]. However, electronic consultation can also offer significant benefits to some groups that are arguably disadvantaged in traditional models of healthcare. Telemedicine supplied to rural areas, for example, could dramatically reduce costs incurred in transfer to specialist care and improve speed of access [10].

Health reforms have resulted in the closure of many regions' hospitals. Consequently, patients may be faced with increasing difficulties in accessing healthcare. Along with this trend, rural New Zealand faces the loss of medical personnel. The Ministry of Health telephone pilot, which serves parts of the East Coast District of the North Island, is a pioneering service that aims to address some of these issues. If successful, it may well pave the way for the formation of services utilizing more sophisticated technology.

Public policy will eventually need to address these issues in the longer term, particularly as public health systems move toward greater use of e-health initiatives. This task may require the eventual supply of resources to selected individuals or groups, such as the provision of community Internet kiosks or centers. Or, as Mulholland has suggested, provision might be made through a contact person, such as a community nurse, who has access to the Internet [4].

#### Consumerism

Information technology gives patients access to a wealth of knowledge and information [11]. An informed patient can participate more actively in healthcare decisions. This circumstance may, however, lead to a situation in which providers find themselves faced with more aggressive and demanding patients, who require more time and explanation [5,12]. It may be difficult to meet these needs within the usual length of commentary supplied in e-mailed responses. It may also be time-consuming to compile and find additional information to attach as an accompanying file or document.

E-mail consultation services may be designed for patients who have an established relationship with a provider or, alternatively, may be offered as a means of attracting business. Egger suggested that when patients indicated that access to their doctor by e-mail was important to them, then doctors would consider introducing e-mail into their practices [13]. Hence, it is feasible that offering this service could give future competitive advantage to a health practice.

The Internet has opened up new opportunities for financial gain. In the space of only a few years there has been a burgeoning number of both small and large e-health providers responding to the demands of a new wave of consumer-driven healthcare seekers. It is now possible for healthcare suppliers to create revenue regardless of physical location and even to offer niche services on a global basis.

#### Provider-Patient Relationships

The extent to which consultation over the Internet will change the patient-provider relationship is unclear. Stevens compared the social impact of the Industrial Revolution with that of information technology [14]. Just as the Industrial Revolution ultimately re-ordered traditional relationships, such as how children related to parents or men related to women, the Internet may likewise radically redefine traditional models. It seems likely that the evolution of styles and frameworks will be one response to the many aspects that communication technology brings to the context.

Historically, a patient base comprised those who lived or worked near a health practice. The Internet now provides the healthcare seeker with the opportunity to decide where to get information. This information may be from a provider far from the patient's locality. A patient may even approach a provider from a different country who is considered to be a leading expert within a particular field [15].

The adoption of e-mail consultation is by no means the only factor that will influence how patients and practitioners interact in the future. Other forces that will have an impact on this interaction include the vast array of information available through Web sites; the increasing financial imperatives to contain costs; and the new generations of software and hardware that enable increasingly sophisticated systems of interaction, for example, SendTalk, PowerTalk, and video-streaming through NetMeeting.

When entering into an e-mail consultation with an unknown online provider, patients will need to take on more responsibility for their treatment. Without the usual tangible evidence that bricks and mortar supply, patients will potentially be exposed to more risk and will have to invest extra time and effort into researching questions to ask providers, assessing quality of responses, and coordinating their own healthcare [16]. "Surfing" providers and the use of advice in a piecemeal fashion also pose significant risks to patients. Currently, it seems unlikely that a provider would happily become involved in cases where multiple consultation and treatment trial are being undertaken, but in the future this practice might be normal.

#### Legal and Ethical Issues

At present there is no special legislation in New Zealand that covers electronic consultation. E-commerce laws are currently being finalized. These laws will be of a general nature and will need to be adapted to cover e-health [17].

The Health Act (1996) and Privacy Act (1993) are deemed to cover this area . The Privacy Act is intended to promote and protect individual privacy in accordance with the Organisation for Economic Co-operation and Development (OECD) Guidelines (1980). Legislation relevant to the health sector is the Health Information Privacy Code (1994; http://www.privacy.org.nz/comply/hinfopc.html), which contains 12 rules regarding the use and disclosure of health information. The code addresses patient empowerment and informed consent. Four rules relevant to electronic consultation are Rules 3, 5, 10, and 11.

Rule 3 requires that the consumer be fully informed about the fact that personal information is being collected. It seems that adherence to this rule is quite poor. Although no survey has been conducted to ascertain the level of compliance with the Privacy Act by New Zealand-based Web sites, an examination by the US Federal Trade Commission of over 1400 Web sites found that although more than 85% of sites collected personal information from consumers, only 2% provided a comprehensive privacy policy [20]. Privacy Commissioner Bruce Sloan has warned that he is prepared to act on any breach of privacy under this code [21], although he would seem to prefer the introduction of self-regulation [22], despite the US Federal Trade Commission's conclusion that "industry's efforts to encourage voluntary adoption of the most basic fair information practice . . . have fallen far short of what is needed to protect consumers" [20].

Rule 5 deals with security and storage and therefore has particular relevance for electronic information. Some of the areas covered are password protection, screensavers, access control, and secure Intranets. Rules 10 and 11 limit health information use and disclosure. There would be interesting implications if browsing were considered "use" of information. The privacy commissioner has deemed that to constitute "use," data must be retrieved and some action must be taken.

On an international level, several non-profit organizations that aim to ensure ethical use of medical information on the Internet have been established. These organizations include the Internet Healthcare Coalition (http://www.ihc.net/) and the Health On the Net Foundation (http://www.hon.ch/). The former has drafted, via the e-Health Ethics Summit, the International e-Health Code of Ethics, and the latter has elaborated a code of conduct for medical and health Web sites. These codes cover issues of quality, privacy, informed consent, and confidentiality, as well as advertising, editorial policy, sponsorship, and authorship. The vehicle for implementing the code has yet to be decided, although various labeling techniques are under development using both cybermetrics and human ratings systems [23,24] .

## Conclusions

The speed of technological development and the eventual public acceptance of it are difficult to wage. In general, development and acceptance are increasing daily. Some people will embrace the advances, others will be hesitant, and still others will disapprove of them. Electronic consultation and e-health are no different in this respect. It seems the global reach of the electronic arm will always ensure a market. What remains variable, for e-health as for healthcare in general, are the issues of quality, accessibility, and confidentiality. In recent years these issues have been addressed in depth by health and legal organizations. The results provide a good existing framework on which to build, and it is in this area that the challenges lie for the next decade.

E-health has its own specific and special aspects related to the doctor-patient relationship and confidentiality. Some we are aware of, others we will encounter as we go. This dynamism characterizes the Internet. Control needs to be flexible and manageable; otherwise, it is open to problems.

New Zealand is known for its early acceptance of new technologies, and our research reflects this tendency. "Dr Global" (http://www.doctorglobal.com/) is here-we expect that more will follow. Yet this change does not signal the demise of face-to-face consultations. There is a place for both services. The challenge is to help make it work for the benefit of all.

## Appendix 1

### URLs Accessed

New Zealand perspectives:

http://www.doctorglobal.com/

http://www.enigma.co.nz/hcro_articles/9810/vol2no12_001.htm

http://www.nzhealth.co.nz/nzdoc/archives.html

http://www.cnn.com/2000/TECH/computing/04/25/nz.doctor.idg/index.html

http://www.xtra.co.nz/homepage/health/main/0,1439,Health%3AAsk+the+Expert%3A,00.html

Other perspectives:

http://www.telemedtoday.com/

http://www.telehealthmag.com/

http://www.yi.com/mednet99/index.htm

http://www.askyourdoctoronthenet.com/

http://www.healthfile.co.uk/

http://www.hon.ch/Conduct.html

http://www.mdweb.com/

http://www.la-doctor.com/main-directory.htm

http://www.marketadoctor.com/index.html

http://www.retina-doctor.com/namequery.htm

http://207.198.253.192/default.htm

http://www.ppdnet.com/content/netdisc/doctorcom.htm

http://www.1-800-doctors.com/index.cfm

http://www.e-med.co.uk/home.html

http://www.dis.port.ac.uk/ndtm/

http://www.ihealthcoalition.org/community/join.html

http://www.atmeda.org/news/testimony04112000.htm

http://www.cyberdialogue.com/resource/press/releases/1999/11-03-cch-ehealth.html

http://www.dc.com/deloitte_research/featured/e-health/e-health.pdf

http://tie.telemed.org/legal/

http://telehealth.net/

http://www.doctorgeorge.com/consultation_room/index.htm

http://www.doctors.net.uk/

http://www.nap.edu/html/networking_health/ch2.html

http://intel.com/intel/e-health/whatisehealth.htm

http://intel.com/intel/e-health/tips.htm

http://intel.com/pressroom/kits/events/9810ihd.htm

http://www.noie.gov.au/projects/ecommerce/ehealth/rise_of_ehealth/ehealth3.htm

http://psychological.com/

http://www.dr-ann.org/
